# Differential Cardiotoxicity of Ibrutinib Versus Chemoimmunotherapy in Chronic Lymphocytic Leukemia: A Population-Based Study

**DOI:** 10.3390/jcm13237492

**Published:** 2024-12-09

**Authors:** Abdulrahman Majrashi, Ying X. Gue, Alena Shantsila, Stella Williams, Gregory Y. H. Lip, Andrew R. Pettitt

**Affiliations:** 1 Liverpool Centre for Cardiovascular Science at University of Liverpool, Liverpool John Moores University and Liverpool Heart & Chest Hospital, Liverpool, UK; s.shantsila@liverpool.ac.uk (A.S.); gregory.lip@liverpool.ac.uk (G.Y.H.L.); 2 Department of Emergency Medical Services, College of Nursing & Health Sciences, Jazan University, Jazan 45142, Saudi Arabia; 3 Department of Cardiovascular and Metabolic Medicine, Institute of Life Course and Medical Sciences, University of Liverpool, Liverpool L69 7ZX, UK; 4 Clatterbridge Cancer Centre NHS Foundation Trust, Liverpool L7 8YA, UK; stella.williams1@nhs.net; 5 Danish Centre for Health Services Research, Department of Clinical Medicine, Aalborg University, 9220 Aalborg, Denmark; 6 Department of Molecular & Clinical Cancer Medicine, University of Liverpool, Liverpool L69 7ZX, UK; arp@liverpool.ac.uk

**Keywords:** ibrutinib, chemoimmunotherapy, cardiovascular toxicities, atrial fibrillation, hypertension

## Abstract

**Background:** Chronic lymphocytic leukaemia (CLL) is the most common form of leukaemia among adults, particularly in Western nations. The introduction of Bruton’s tyrosine kinase (BTK) inhibitors as a treatment of CLL, namely, ibrutinib, which is a first-generation BTK inhibitor, has significantly improved the treatment landscape for CLL. However, ibrutinib has been associated with an increased risk of atrial fibrillation (AF) and hypertension. Real-world studies that compare the cardiovascular safety of ibrutinib with bendamustine plus anti-CD20 monoclonal antibody are not widely available. **Methods:** A retrospective cohort analysis using the TriNetX platform identified two patient groups: one treated with ibrutinib and the other with bendamustine and an anti-CD20 antibody. Propensity score matching balanced their demographic and clinical characteristics. The outcomes evaluated included the all-cause mortality and new-onset AF/flutter, hypertension, heart failure, ventricular arrhythmias, and bleeding. **Results:** No significant difference was observed in the all-cause mortality between the two cohorts. However, ibrutinib was associated with a higher risk of AF/flutter (HR 1.89, 95% CI 1.36–2.62; *p* < 0.05) and hypertension (HR 1.22, 95% CI 1.01–1.47; *p* = 0.04). Other outcomes, such as heart failure, ventricular arrhythmias, and bleeding, were not different between the cohorts. **Conclusions:** Ibrutinib remains a valuable option for the treatment of CLL, but is associated with significant cardiovascular risks, leading to it being superseded by the newer generation of BTKis, which offer less cardiovascular toxicities. These results highlight the TriNetX platform’s reliability as a real-world data source for validating clinical trial findings and highlight the importance of incorporating cardio-oncology into treatment plans for CLL patients with significant comorbidities.

## 1. Introduction

Chronic lymphocytic leukaemia (CLL) is the most prevalent form of leukaemia in adults, with incidence rates of 4 to 6 cases per 100,000 people annually in Western nations. The disease predominantly affects older adults. Small lymphocytic lymphoma (SLL), although distinct in its presentation, shares a similar pathophysiology with CLL [1]. Until recently, due to its efficacy in most patients, CLL treatment for patients over 65 years old involved chemo(immuno)therapy, such as bendamustine combined with an anti-CD20 monoclonal antibody, such as rituximab [2]. However, recent insights into the pathophysiology of CLL have led to the emergence of effective novel targeted therapies [3].

The treatment landscape for CLL has evolved dramatically with the introduction of Bruton’s tyrosine kinase (BTK) inhibitors. Ibrutinib was the first BTK inhibitor (BTKi) approved for the treatment of CLL. It suppresses and blocks BTK enzyme activity by covalently binding to the same ATP domain [4,5], which significantly improves long-term outcomes for untreated or relapsed/refractory CLL patients in phase 3 trials when compared to chemo(immuno)therapy alone or in combination [6,7]. However, treatment with ibrutinib has been commonly associated with an increased risk of several adverse cardiovascular events, including atrial fibrillation (AF) and hypertension [8,9], with some concerns regarding an increased risk of heart failure and ventricular arrhythmias [9].

As bendamustine and rituximab might be a tolerable alternative for elderly patients who are high-risk for cardiovascular adverse events [10], and in some developing low- and middle-income countries, novel agents could be limited to routine practice due to several reasons such as cost and a lack of regulatory approvals [11,12]; for example, according to market data in India, not all patients eligible for ibrutinib are treated with the drug [13].

This study aims to address and compare the risk of adverse effects between the two treatment options by using sizable real-world data.

## 2. Method

### 2.1. TriNetX Platform

This study was a retrospective cohort analysis using the TriNetX (TriNetX, Cambridge, MA, USA) platform, a global health research network integrating de-identified electronic medical records (EMRs) from numerous healthcare organisations. The platform provides comprehensive patient data, including demographics, clinical diagnoses, procedures, medications, and laboratory results, enabling a detailed evaluation of real-world clinical outcomes. Additional details about the TriNetX platform can be found online at https://open.trinetx.com/company-overview/ (accessed on 1 November 2024).

### 2.2. Patient Selection

The study search included data from 128 healthcare organisations within the Global Collaborative Network of TriNetX. Two distinct cohorts were created based on patients’ treatment regimens. Cohort 1 consisted of patients treated with ibrutinib (RXNORM: 1442981), while cohort 2 comprised patients treated with bendamustine (RXNORM: 134547) in combination with any anti-CD20 monoclonal antibody (rituximab, obinutuzumab, or ofatumumab). Patients in both cohorts were aged 18 years or older and had been diagnosed with B-cell CLL or small-cell B-cell lymphoma based on ICD-10 codes. Patients with pre-existing AF, hypertension, or heart failure and conflicted treatments with both agents before the index treatment were excluded to minimise confounding by pre-existing cardiovascular conditions and treatments. Furthermore, RECORD-PE reporting guidelines have been adhered to [14], with the checklist provided in the Supplementary Materials.

### 2.3. Propensity Score Matching (PSM)

PSM was used to ensure a balanced comparison between the two cohorts, matching the patients based on key demographic and clinical variables, including demographics (age, sex, and ethnicity), cardiovascular and cerebrovascular diseases (ischemic heart diseases, pulmonary embolism, and cerebral infarction), risk factors for bleeding (abnormal coagulation profile), cardiovascular diseases (nicotine dependence, diabetes mellitus, obesity, lipid disorder, and chronic kidney diseases), and cardiovascular procedures (see Table S2). After performing the matching process, standardised difference was obtained to evaluate the balance of patient characteristics between the matched cohorts. A standardised difference below 0.1 indicates that the groups are well balanced.

### 2.4. Outcome Measurement

The primary outcome was all-cause death, defined as the time from treatment initiation to death from any cause following three years. Secondary outcomes included new-onset AF/flutter, hypertension, heart failure, ventricular arrhythmias, and bleeding.

### 2.5. Statistical Analyses

All statistical analyses were conducted using the TriNetX platform integrated tool (accessed on 23 August 2024), which includes R survival package v3.2-3. For baseline characteristics, categorical variables were compared between the two cohorts using the chi-square test, while *t*-test was considered to evaluate continuous variables. Longitudinal outcomes were analysed using Kaplan–Meier survival analyses performed to compare outcomes. Hazard ratios (HRs) with 95% confidence intervals (CIs) were calculated using Cox proportional hazards models. Statistical significance was determined with a *p*-value at <0.05.

## 3. Results

The initial search found 2704 patients treated with ibrutinib and 1075 patients treated with bendamustine in combination with an anti-CD20 monoclonal antibody, mainly rituximab, from 72 different healthcare institutions. Following PSM, each cohort consisted of 977 patients. The cohorts were well balanced regarding key demographic and clinical characteristics, ensuring a valid comparison. The mean age of the patients in both cohorts was approximately 66 years, with a slight predominance of males, reflecting the typical demographic profile of CLL patients. All characteristics, including the body mass index, smoking status, and medical history, were evenly distributed between the cohorts (Table 1).

## 4. Primary and Secondary Outcomes

**Figure 1:**
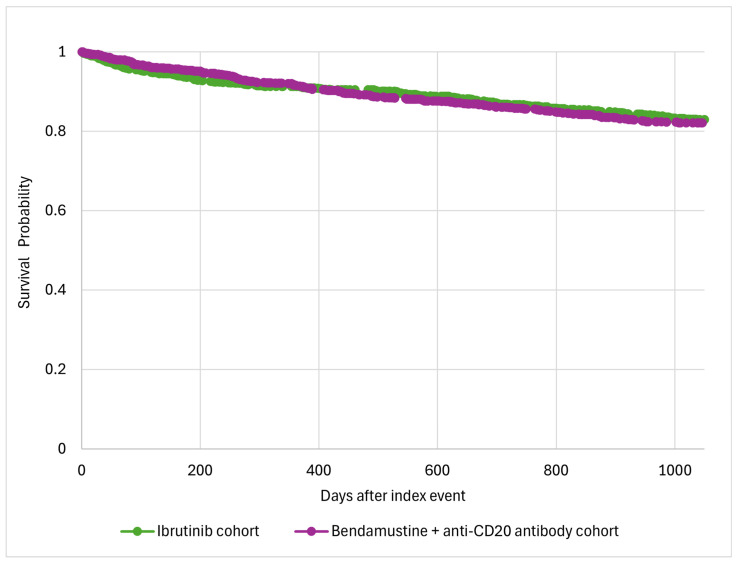
Kaplan–Meier survival analysis.

The analysis revealed no significant difference in the all-cause mortality between the two cohorts (15.5% vs. 16.3%; HR 0.94 [95% CI 0.75–1.17]; *p*= 0.6) (Table 2, Figure 1). However, the patients in the ibrutinib cohort showed a significantly higher risk of developing AF/flutter (10.3% vs. 5.6%; HR 1.88 [95% CI 1.35–2.62]; *p* < 0.05) and hypertension (23.5% vs. 19.9%; HR 1.21 [95% CI 1.00–1.47]; *p* = 0.04) compared to the patients treated with bendamustine plus an anti-CD20 monoclonal antibody. In contrast, the risks of heart failure (1.7% vs. 1.8%; HR 0.94 [95% CI 0.48–1.83]; *p* = 0.8), ventricular arrhythmias (1.5% vs. 1.0%; HR 2.15 [95% CI 0.87–5.29]; *p* = 0.09), and bleeding (8.5% vs. 7.5%; HR 1.14 [95% CI 0.83–1.56]; *p* = 0.4) did not differ significantly between the treatment groups (Table 2).

## 5. Discussion

By using a sizable population-level real-world dataset, our study showed that ibrutinib contributes to a greater risk of AF and hypertension compared to bendamustine in combination with an anti-CD20 monoclonal antibody, and in doing so, confirms the results of the Alliance US trial in a large real-world setting.

This study offers a valuable perspective into a comparative safety profile of ibrutinib and bendamustine plus an anti-CD20 monoclonal antibody among patients with CLL/SLL, focusing on all causes of death, cardiovascular outcomes, and bleeding events.

In this study, all the causes of death were not different between the ibrutinib and bendamustine plus anti-CD20 antibodies cohorts (15.5% vs. 16.3%). In keeping with this, the results of the US Alliance trial showed no difference in the overall survival among the CLL patients treated with ibrutinib or bendamustine combined with rituximab at 90% and 95%, respectively, at two years [7].

Our study shows a higher incidence of AF/flutter among the CLL/SLL patients treated with ibrutinib compared to the combination of bendamustine and anti-CD20 antibodies (10.3% vs. 5.6%). These results are consistent with the trial comparing the two therapeutic options where the incidence of any-grade AF associated with ibrutinib in comparison to bendamustine plus rituximab was reported as 17% vs. 3%, and for grade 3, 8% vs. 3%), respectively [7]. A recent meta-analysis reported that BTKi including newer generation agents, are associated with a higher risk of AF compared to chemoimmunotherapy [15]. Furthermore, a real-world study showing that, upon comparing CLL patients that were exposed and not exposed to ibrutinib, in general, the patients who were exposed to ibrutinib developed AF [16], and the same observation was noticed in another real-world analysis evaluating cardiovascular events in CLL patients, which found that AF was more frequently observed in those receiving ibrutinib monotherapy compared to intensive therapy. Moreover, among patients not treated with intensive regimens, ibrutinib was also associated with a higher risk of AF [17].

The underlying mechanisms of BTKi-induced AF remain unknown, but there are multiple potential explanations. BTKi-induced AF could be related to wider selectivity of ibrutinib inhibition by the BTK enzyme, and could lead to off-target effects as a result of binding to kinases other than the BTK enzyme [18]. Incidents of AF were observed less with newer generations of BTKis, namely, acalabrutinib [19] or zanbrutinib, when compared with ibrutinib among patients with B-cell malignancies [20,21], which have a greater selectivity for the BTK enzyme [18]. Still, despite the lower incidence of AF associated with newer-generation BTKis compared to ibrutinib, Tarnowski et al. found that ibrutinib and acalabrutinib showed comparable effects on calcium handling in cultured myocytes treated with IGF-1, reducing the calcium transient amplitude and slowing its decay [22].

In the preclinical setting, ibrutinib is shown to suppress human epidermal growth factor 2, which is known as a key component in maintaining cardiomyocytes haemostasis [23,24]. Furthermore, Xiao et al. found that atrial myocytes differ from other cells of cardiac muscle by the expression of C-terminal kinase (CSK). The suppression and inhibition of CSK has been shown to contribute to ibrutinib-induced AF by causing inflammation and fibrosis of the atria in animal models [25]. Given the thromboembolic risk that could arise from AF and bleeding associated with ibrutinib treatment in CLL patients [9,26,27], the management of these conditions may require closely coordinated treatment plans developed through collaboration between haemato-oncologists and cardiologists. Lyon, Leszek-Fernandez et al. suggest that non-vitamin K antagonist oral anticoagulants are the preferred choice for anticoagulation in cardio-oncology patients with AF, except in cases where contraindications are present [26].

Similarly, in terms of hypertension, our study has shown that 23.5% of the patients receiving ibrutinib developed new-onset hypertension compared to 19.9% in the bendamustine plus anti-CD20 antibody cohort, which is reflective of the current literature findings [7,28]. The same observation was noted in the US Alliance trial, where 29% and 14% of the CLL patients who received ibrutinib and bendamustine in combination with rituximab experienced grade 3 or higher hypertension, respectively [7]. It is worth noting that the rate of hypertension in the trial was relatively high, possibly due to including CLL patients age 65 years or older, where the prevalence of hypertension increases approximately by 75% for individuals aged 60 years or over in a different setting [29], and among CLL patients with advanced age treated with ibrutinib [30].

The mechanism(s) underlying ibrutinib-induced hypertension is unknown but is likely related to endothelial dysfunction, where the inhibition of BTK has been shown to interfere with nitric oxide synthesis [31], leading to increased vascular resistance and hypertension. Furthermore, cellular remodelling may lead to the fibrosis of vascular tissue due to the suppression of the PI3K pathway [32], whereas advanced age could lead to several physiological changes, including arterial stiffness, endothelial dysfunction, and reduced renal capacity, leading to fluid retention and increased vascular resistance. Additionally, reduction in the baroreceptor sensitivity further impairs blood pressure regulation, all of which could contribute to sustained hypertension [33]. Furthermore, some concerns have been raised that bendamustine in combination with rituximab may contribute to vascular toxicity and nephrotoxicity [34]. Despite the presence of a significantly higher incidence of hypertension among the patients treated with ibrutinib in our study, the treatment with bendamustine in combination with rituximab could be a suitable therapeutic option among elderly CLL patients when other alternatives are extremely limited, such as the unavailability of novel agents and lack of approval in certain countries [35,36]. ACE inhibitors or ARBs are often recommended as first-line treatments for hypertension associated with cancer therapies. In case of stage 2 hypertension, combining these agents with calcium channel blockers could be an appropriate approach [26], and close monitoring for any signs of hypertension is crucial during treatment.

The incidence of heart failure and ventricular arrhythmias were similar between the two cohorts in this study, at 1.7% vs. 1.8% and 1.5% vs. 1.0% for ibrutinib and bendamustine plus anti-CD20 antibodies. Despite the lack of rigorous evidence about ibrutinib-induced heart failure or ventricular arrhythmias, growing concerns have been noted in multiple studies [9,16,37,38,39]; further, evidence about bendamustine plus rituximab has shown that cardiomyopathy and heart failure has been observed [40,41]. Consequently, in our study, no difference was noted between the two cohorts that could be attributed to the risk of heart failure associated with chemoimmunotherapy. In terms of ventricular arrhythmias, rituximab has been associated with cardiac arrhythmias including ventricular arrhythmias, particularly during drug infusion [42,43].

This study’s findings revealed comparable bleeding rates between the ibrutinib group (8.5%) and bendamustine plus anti-CD20 antibody group (7.5%). Bleeding is not an uncommon complication of ibrutinib; a study has shown that major bleeding increases by 7.5-fold with ibrutinib compared to other regimens of CLL treatment [27]. On the other hand, the findings of the US Alliance trial showed that grade 3 or higher bleeding incidents were recorded in 2% of the patients on ibrutinib-based therapies, while none were reported in the bendamustine combined with rituximab group [7]. Notably, in the Alliance trial prior to enrolment, the patients were required to stop taking vitamin K antagonists and warfarin, which could explain the slightly higher incidences of bleeding in our study.

The mechanism behind bleeding in ibrutinib treatment is linked to the inhibition of platelet aggregation via GPVI receptor interference, resulting in platelet dysfunction and an increased risk of bleeding [44]. On the other hand, bleeding associated with bendamustine therapy is due to myelosuppression and thrombocytopenia, leading to reduced platelet counts [45]. Despite these distinct pathways, platelet dysfunction with ibrutinib and thrombocytopenia with bendamustine, the overall bleeding risk remains similar between the two treatments, likely due to the role of careful clinical management, including regular monitoring and dose modifications, which help prevent severe bleeding events. When this study was conducted, the ICD-10 codes used to capture bleeding incidents mostly represented major bleeding (Supplementary Materials); therefore, the incidences of major bleeding did not differ.

### Limitations

Our study contains several limitations. These include the retrospective design, which could impose selection bias; the PSM analysis, which does not consider unmeasured confounding factors such as prognostic factors for CLL; and the adherence with BTKi therapy. The data reliability cannot be totally accurate due to unavoidable common human mistakes such as incomplete and missing data or coding errors. Additionally, excluding patients with pre-existing cardiovascular conditions may limit the generalisability of the findings to a broader CLL population. Furthermore, this analysis may be influenced by residual confounding, time-dependent confounding, and competing risk. Moreover, the possibility of type I and type II errors should be acknowledged. For example, the lack of precision in the estimate for ventricular arrhythmias indicates that the analysis for this outcome might lack sufficient statistical power. Nonetheless, this study offers evidence about ibrutinib about cardiovascular adverse events in a sizable real-world CLL population, validating the evidence of the US Alliance trial and providing a direct comparison between ibrutinib against chemoimmunotherapy.

In conclusion, while ibrutinib remains a valuable option for the treatment of CLL, it is associated with significant cardiovascular risks, leading to it being superseded by the newer generation of BTKis, which offer less cardiovascular toxicities. These findings highlight the importance of multidisciplinary collaboration between haemato-oncologists and cardiologists when the indications and treatment decisions are profound, especially for high-risk patients with significant comorbidities.

## Figures and Tables

**Table 1:** Baseline characteristics, medications, and diagnoses before and after propensity score matching.

Characteristic	Before PSM Ibrutinib (*n* = 2704)	Before PSM B+ Anti CD20 (*n* = 1075)	Standardised Difference	After PSM Ibrutinib (*n* = 977)	After PSM B+ Anti CD20 (*n* = 977)	Standardised Difference
Age at Index (Mean ± SD)	67.5 ± 10.8	65.5 ± 10.7	0.183	66.0 ± 10.6	65.7 ± 10.4	0.027
Male	1729 (63.9%)	657 (61.1%)	0.017	609 (62.3%)	601 (61.5%)	0.017
Female	975 (36.1%)	418 (38.9%)	0.058	368 (37.7%)	376 (38.5%)	0.017
White	2117 (78.3%)	778 (72.4%)	0.138	716 (73.3%)	720 (73.7%)	0.009
Black or African American	132 (4.9%)	41 (3.8%)	0.052	39 (4%)	39 (4%)	<0.001
Hepatomegaly and splenomegaly	331 (12.2%)	238 (22.1%)	0.265	188 (19.2%)	196 (20.1%)	0.021
Abnormal coagulation profile	10 (0.4%)	11 (1%)	0.079	10 (1%)	10 (1%)	<0.001
Personal history of antineoplastic chemotherapy	133 (4.9%)	72 (6.7%)	0.076	51 (5.2%)	55 (5.6%)	0.018
Heart failure	0 (0%)	10 (0.9%)	0.137	0 (0%)	0 (0%)	--
Diabetes mellitus	180 (6.7%)	79 (7.3%)	0.027	59 (6%)	70 (7.2%)	0.045
Hypertensive diseases	0 (0%)	41 (3.8%)	0.282	0 (0%)	0 (0%)	--
AF/flutter	0 (0%)	10 (0.9%)	0.137	0 (0%)	0 (0%)	--
Overweight, obesity, and other hyperalimentation	137 (5.1%)	58 (5.4%)	0.015	49 (5%)	51 (5.2%)	0.009
Disorders of lipoprotein metabolism	497 (18.4%)	210 (19.5%)	0.029	180 (18.4%)	186 (19%)	0.016
Chronic kidney disease (CKD)	101 (3.7%)	58 (5.4%)	0.080	37 (3.8%)	45 (4.6%)	0.041
Ischemic heart diseases	149 (5.5%)	74 (6.9%)	0.057	55 (5.6%)	59 (6%)	0.017
Cerebral infarction	19 (0.7%)	17 (1.6%)	0.083	10 (1%)	11 (1.1%)	0.010
Pulmonary embolism	29 (1.1%)	23 (2.1%)	0.085	17 (1.7%)	19 (1.9%)	0.015
Surgical procedures on the cardiovascular system	1348 (49.9%)	696 (64.7%)	0.305	615 (62.9%)	618 (63.3%)	0.006
Alpha-blockers/related	242 (8.9%)	92 (8.6%)	0.014	90 (9.2%)	87 (8.9%)	0.011
Ace inhibitors	143 (5.3%)	53 (4.9%)	0.016	52 (5.3%)	48 (4.9%)	0.019
Beta blockers/related	319 (11.8%)	150 (14%)	0.064	122 (12.5%)	119 (12.2%)	0.009
Calcium channel blockers	134 (5%)	67 (6.2%)	0.056	36 (3.7%)	44 (4.5%)	0.041
Diuretics	331 (12.2%)	194 (18%)	0.162	141 (14.4%)	151 (15.5%)	0.029
Antiarrhythmics	696 (25.7%)	477 (44.4%)	0.398	397 (40.6%)	404 (41.4%)	0.015
Antilipemic agents	552 (20.4%)	183 (17%)	0.087	139 (14.2%)	161 (16.5%)	0.062
Angiotensin ii inhibitor	109 (4%)	48 (4.5%)	0.022	27 (2.8%)	30 (3.1%)	0.018
Anticoagulants	617 (22.8%)	436 (40.6%)	0.388	373 (38.2%)	360 (36.8%)	0.027
Platelet aggregation inhibitors	415 (15.3%)	196 (18.2%)	0.077	168 (17.2%)	169 (17.3%)	0.003

**Table 2:** Outcomes analysis among two cohorts.

Outcome	Cohort	Patients in Cohort	Patients with Outcome (%)	HR (95% CI)
All-cause death	Ibrutinib	977	151 (15.5%)	0.94 (0.75–1.17)
	B+ anti-CD20 antibody	977	159 (16.3%)	
AF/Flutter	Ibrutinib	977	101 (10.3%)	1.88 (1.35–2.62)
	B+ anti-CD20 antibody	977	55 (5.6%)	
Hypertension	Ibrutinib	977	230 (23.5%)	1.21 (1.00–1.47)
	B+ anti-CD20 antibody	977	194 (19.9%)	
Heart failure	Ibrutinib	977	17 (1.7%)	0.94 (0.48–1.83)
	B+ anti-CD20 antibody	977	18 (1.8%)	
Ventricular arrhythmias	Ibrutinib	977	18 (1.5%)	2.15 (0.87–5.29)
	B+ anti-CD20 antibody	977	10 (1.0%)	
Bleeding	Ibrutinib	977	83 (8.5%)	1.14 (0.83–1.56)
	B+ anti-CD20 antibody	977	73 (7.5%)	

Abbreviations: B, bendamustine; AF, atrial fibrillation; HR, hazard ratio; CI, confidence interval.

## Data Availability

Additional information about the statistical analysis and propensity score matching can be found in the Supplementary Materials.

## Supplementary Materials

The following supporting information can be downloaded at https://www.mdpi.com/article/10.3390/jcm13237492/s1, Table S1: ICD-10-CM codes for primary and secondary outcomes; Table S2: Baseline characteristics for two cohorts before and after propensity score matching.
