# Supplementary material for: Differential Cardiotoxicity of Ibrutinib Versus Chemoimmunotherapy in Chronic Lymphocytic Leukemia: A Population-Based Study
Source: J Clin Med. 2024 Dec 9;13(23):7492. doi: 10.3390/jcm13237492 (PMC11641840; doi:10.3390/jcm13237492)
Supplement: Supplementary file 1 [file jcm-13-07492-s001.zip › jcm-3321631-supplementary.pdf]

Supplementary Materials

Supplementary Methodological Details

The TriNetX network is a research tool widely used for scientific investigations, fully compliant with both the Health Insurance Portability and Accountability Act (HIPAA) and U.S. federal regulations that safeguard healthcare data privacy and security. This includes de-identified data, which is managed according to HIPAA's de-identification standards (https://trinetx.com/real-world-resources/publications/); therefore, no direct consent is required from patients by investigators. Accessing TriNetX data requires submitting a formal request and establishing a data-sharing agreement. As a federated research network, TriNetX facilitates studies without necessitating ethical approval since it provides no direct access to identifiable patient information.

Data within TriNetX is hosted through either physical servers located at institutional data centres or via a virtual appliance. The network operates as a federation of these appliances, each capable of processing queries that are broadcasted and then aggregating the results. Upon integration into the network, all data is standardised to a controlled clinical terminology set and subjected to quality assessment processes. This includes stringent 'data cleaning' protocols that exclude records failing to meet TriNetX quality benchmarks. Each database update undergoes a rigorous quality evaluation based on criteria of conformance, completeness, and plausibility (http://doi.org/10.13063/2327-9214.1244).

To comply with HIPAA standards, patient data is de-identified, with available information including demographics, diagnoses (ICD-10-CM codes), procedures (ICD-10-PCS or CPT codes), and measurements (LOINC codes). Although patient diagnoses and procedures are well-documented, other factors, such as socioeconomic and lifelong exposures, maybe less thoroughly represented. Electronic health records (EHR) offer advantages over data obtained from insurance claims by including both insured and uninsured patients. Compared to survey data, EHRs also offer a more accurate reflection of diagnostic prevalence within healthcare-utilising populations, highlighting the true burden of specific diseases on healthcare systems.

Nonetheless, relying on diagnosis data has its limitations, as undiagnosed conditions remain unaccounted for and may not appear. Moreover, because patients may seek care across multiple organisations, some parts of their records may be missing if any of their healthcare providers do not participate in the TriNetX network. Although a multi-organizational network mitigates this issue, it does not entirely eliminate it.

Propensity Score Matching Analyses

To control for potential confounding, TriNetX uses logistic regression within the Python (version 3.7) scikit-learn package to perform 1:1 greedy nearest-neighbour matching with a calliper of 0.1 pooled standard deviations. To minimise bias from neighbour algorithms, row orders are randomised before matching. Baseline characteristics with standardised mean differences below 0.1 between matched cohorts show that the level of balance is acceptable.

(https://www.tandfonline.com/doi/full/10.1080/00273171.2011.568786).

Table S1. ICD-10-CM codes for primary and secondary outcomes.

**Outcomes**	**ICD-10-CM-codes**
All-cause death	Deceased (variable codified by TriNetX).
Atrial fibrillation	I48 Atrial fibrillation and flutter
Hypertension	I10-I1A Hypertensive disease
Acute Heart failure	I50.21 Acute Systolic heart failure<br>I50.23 Acute on chronic systolic heart failure<br>I50.31 Acute diastolic heart failure<br>I50.33 Acute on chronic diastolic heart failure
Ventricular arrhythmias	I49.0 Ventricular fibrillation and flutter<br>and/or<br>I47.2 Ventricular tachycardia
Bleeding	R58 Hemorrhage, not elsewhere classified<br>and/ or<br>I60 Nontraumatic subarachnoid hemorrhage<br>I61 Nontraumatic intracerebral hemorrhage<br>I62 Other and unspecified Nontraumatic intracranial hemorrhage<br>K92.2 Gastrointestinal hemorrhage, unspecified<br>I60 Nontraumatic subarachnoid hemorrhage<br>K26 Duodenal ulcer

	K27 Peptic ulcer, unspecified<br><br>K62.5 hemorrhage from anus and rectum<br><br>R04.0 Epistaxis<br><br>R04.2 Hemoptysis

Table S2. Baseline characteristics for two cohorts before and after propensity score matching.

Cohort 1 and cohort 2 patient count before and after propensity score matching

Cohort	Patient count before matching	Patient count after matching
1 - Ibrutinib vs B+ anti CD20	2,704	977
2 - B+ anti CD20 vs Ibrutinib	1,075	977

Propensity score density function - Before and after matching (cohort 1 - purple, cohort 2 - green)

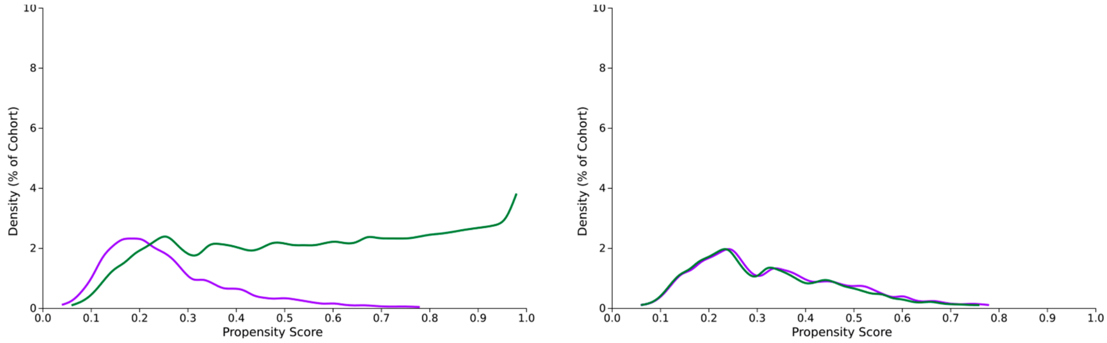

Cohort 1 (N = 2,704) and cohort 2 (N = 1,075) characteristics before propensity score matching

Demographics

Cohort			Mean ± SD	Patients	% of Cohort	P-Value	Std diff.
1<br>2	AI	Age at Index	67.5 +/- 10.8<br>65.5 +/- 10.7	2,704<br>1,075	100%<br>100%	<0.001	0.183
1<br>2	2106-3	White		2,117<br>778	78.3%<br>72.4%	<0.001	0.138
1<br>2	F	Female		975<br>418	36.1%<br>38.9%	0.104	0.058
1<br>2	2054-5	Black or African American		132<br>41	4.9%<br>3.8%	0.157	0.052

Diagnosis

Cohort			Mean ± SD	Patients	% of Cohort	P-Value	Std diff.
1<br>2	R16	Hepatomegaly and splenomegaly, not elsewhere classified		331<br>238	12.2%<br>22.1%	<0.001	0.265

1<br>2	R79.1	Abnormal coagulation profile		10<br>11	0.4%<br>1.0%	0.015	0.079
1<br>2	Z92.21	Personal history of antineoplastic chemotherapy		133<br>72	4.9%<br>6.7%	0.029	0.076
1<br>2	I50	Heart failure		0<br>10	0%<br>0.9%	<0.001	0.137
1<br>2	E08-E13	Diabetes mellitus		180<br>79	6.7%<br>7.3%	0.447	0.027
1<br>2	I10-I1A	Hypertensive diseases		0<br>41	0%<br>3.8%	<0.001	0.282
1<br>2	I48	Atrial fibrillation and flutter		0<br>10	0%<br>0.9%	<0.001	0.137
1<br>2	E65-E68	Overweight, obesity and other hyperalimentation		137<br>58	5.1%<br>5.4%	0.680	0.015
1<br>2	E78	Disorders of lipoprotein metabolism and other lipidemias		497<br>210	18.4%<br>19.5%	0.412	0.029
1<br>2	N18	Chronic kidney disease (CKD)		101<br>58	3.7%<br>5.4%	0.022	0.080
1<br>2	I20-I25	Ischemic heart diseases		149<br>74	5.5%<br>6.9%	0.106	0.057
1<br>2	I63	Cerebral infarction		19<br>17	0.7%<br>1.6%	0.012	0.083
1<br>2	I26	Pulmonary embolism		29<br>23	1.1%<br>2.1%	0.011	0.085
**Procedure**							
Cohort			Mean ± SD	Patients	% of Cohort	P-Value	Std diff.
1<br>2	1006056	Surgical Procedures on the Cardiovascular System		1,348<br>696	49.9%<br>64.7%	<0.001	0.305
**Medication**							

Cohort			Mean ± SD	Patients	% of Cohort	P-Value	Std diff.
1<br>2	CV150	ALPHA BLOCKERS/RELATED		242<br>92	8.9%<br>8.6%	0.702	0.014
1<br>2	CV800	ACE INHIBITORS		143<br>53	5.3%<br>4.9%	0.654	0.016
1<br>2	CV100	BETA BLOCKERS/RELATED		319<br>150	11.8%<br>14.0%	0.070	0.064
1<br>2	CV200	CALCIUM CHANNEL BLOCKERS		134<br>67	5.0%<br>6.2%	0.115	0.056
1<br>2	CV700	DIURETICS		331<br>194	12.2%<br>18.0%	<0.001	0.162
1<br>2	CV300	ANTIARRHYTHMICS		696<br>477	25.7%<br>44.4%	<0.001	0.398
1<br>2	CV350	ANTILIPEMIC AGENTS		552<br>183	20.4%<br>17.0%	0.018	0.087
1<br>2	CV805	ANGIOTENSIN II INHIBITOR		109<br>48	4.0%<br>4.5%	0.546	0.022
1<br>2	BL110	ANTICOAGULANTS		617<br>436	22.8%<br>40.6%	<0.001	0.388
1<br>2	BL117	PLATELET AGGREGATION INHIBITORS		415<br>196	15.3%<br>18.2%	0.030	0.077

Cohort 1 (N = 977) and cohort 2 (N = 977) characteristics after propensity score matching

Demographics

Cohort			Mean ± SD	Patients	% of Cohort	P-Value	Std diff.
1<br>2	AI	Age at Index	66.0 +/- 10.6<br>65.7 +/- 10.4	977<br>977	100%<br>100%	0.555	0.027
1<br>2	2106-3	White		716<br>720	73.3%<br>73.7%	0.838	0.009
1<br>2	F	Female		368<br>376	37.7%<br>38.5%	0.709	0.017

1<br>2	2054-5	Black or African American		39<br>39	4.0%<br>4.0%	1	<0.001
**Diagnosis**							
Cohort			Mean ± SD	Patients	% of Cohort	P-Value	Std diff.
1<br>2	R16	Hepatomegaly and splenomegaly, not elsewhere classified		188<br>196	19.2%<br>20.1%	0.649	0.021
1<br>2	R79.1	Abnormal coagulation profile		10<br>10	1.0%<br>1.0%	1	<0.001
1<br>2	Z92.21	Personal history of antineoplastic chemotherapy		51<br>55	5.2%<br>5.6%	0.690	0.018
1<br>2	I50	Heart failure		0<br>0	0%<br>0%	--	--
1<br>2	E08-E13	Diabetes mellitus		59<br>70	6.0%<br>7.2%	0.316	0.045
1<br>2	I10-I1A	Hypertensive diseases		0<br>0	0%<br>0%	--	--
1<br>2	I48	Atrial fibrillation and flutter		0<br>0	0%<br>0%	--	--
1<br>2	E65-E68	Overweight, obesity and other hyperalimentation		49<br>51	5.0%<br>5.2%	0.837	0.009
1<br>2	E78	Disorders of lipoprotein metabolism and other lipidemias		180<br>186	18.4%<br>19.0%	0.728	0.016
1<br>2	N18	Chronic kidney disease (CKD)		37<br>45	3.8%<br>4.6%	0.367	0.041
1<br>2	I20-I25	Ischemic heart diseases		55<br>59	5.6%<br>6.0%	0.699	0.017
1<br>2	I63	Cerebral infarction		10<br>11	1.0%<br>1.1%	0.826	0.010
1<br>2	I26	Pulmonary embolism		17<br>19	1.7%<br>1.9%	0.737	0.015

Procedure							
Cohort			Mean ± SD	Patients	% of Cohort	P-Value	Std diff.
1<br>2	1006056	Surgical Procedures on the Cardiovascular System		615<br>618	62.9%<br>63.3%	0.888	0.006

Medication							
Cohort			Mean ± SD	Patients	% of Cohort	P-Value	Std diff.
1<br>2	CV150	ALPHA BLOCKERS/RELATED		90<br>87	9.2%<br>8.9%	0.813	0.011
1<br>2	CV800	ACE INHIBITORS		52<br>48	5.3%<br>4.9%	0.681	0.019
1<br>2	CV100	BETA BLOCKERS/RELATED		122<br>119	12.5%<br>12.2%	0.836	0.009
1<br>2	CV200	CALCIUM CHANNEL BLOCKERS		36<br>44	3.7%<br>4.5%	0.361	0.041
1<br>2	CV700	DIURETICS		141<br>151	14.4%<br>15.5%	0.526	0.029
1<br>2	CV300	ANTIARRHYTHMICS		397<br>404	40.6%<br>41.4%	0.747	0.015
1<br>2	CV350	ANTILIPEMIC AGENTS		139<br>161	14.2%<br>16.5%	0.167	0.062
1<br>2	CV805	ANGIOTENSIN II INHIBITOR		27<br>30	2.8%<br>3.1%	0.687	0.018
1<br>2	BL110	ANTICOAGULANTS		373<br>360	38.2%<br>36.8%	0.544	0.027
1<br>2	BL117	PLATELET AGGREGATION INHIBITORS		168<br>169	17.2%<br>17.3%	0.952	0.003
